# Design of 2 μm Low-Loss Hollow-Core Anti-Resonant Fibers

**DOI:** 10.3390/mi14061198

**Published:** 2023-06-05

**Authors:** Tianran Sun, Xinyang Su, Fanchao Meng, Zaining Wang, Jiale Song, Chenglong Zhang, Tianjia Xu, Yunhong Zhang, Huaiwei Zhang, Mengdi Cui, Yi Zheng

**Affiliations:** 1 School of Physical Science and Engineering, Beijing Jiaotong University, Beijing 100044, China; 18118043@bjtu.edu.cn (T.S.); mfanchao@126.com (F.M.); 20271163@bjtu.edu.cn (Z.W.); 20271044@bjtu.edu.cn (J.S.); 21271278@bjtu.edu.cn (C.Z.); 19118047@bjtu.edu.cn (Y.Z.); 18126233@bjtu.edu.cn (H.Z.); 21118043@bjtu.edu.cn (M.C.); 2 Key Laboratory of Luminescence and Optical Information, Ministry of Education, Beijing Jiaotong University, Beijing 100044, China; 3 School of Optics and Photonics, Beijing Institute of Technology, Beijing 100081, China; 3220220556@bit.edu.cn

**Keywords:** anti-resonant fiber (ARF), higher-order mode extinction ratio (HOMER), confinement loss (CL)

## Abstract

We systematically studied several of the most traditional hollow-core anti-resonant fiber (HC-ARF) structures, with the aim of achieving low confinement loss, single-mode performance, and high insensitivity to bending in the 2 µm band. Moreover, the propagation loss of fundamental mode (FM), higher-order mode (HOMs), and the higher-order mode extinction ratio (HOMER) under different geometric parameters were studied. Analysis showed that the confinement loss of the six-tube nodeless hollow-core anti-resonant fiber at 2 µm was 0.042 dB/km, and its higher-order mode extinction ratio was higher than 9000. At the same time, a confinement loss of 0.040 dB/km at 2 µm was achieved in the five-tube nodeless hollow-core anti-resonant fiber, and its higher-order mode extinction ratio was higher than 2700.

## 1. Introduction

Recent years have seen the development of hollow-core fibers. Due to the extraordinary light-guiding properties of hollow-core fibers, they have attracted extensive research and manufacture by research groups around the world [1,2,3,4,5,6,7,8]. Most of the energy (>99.99%) can be concentrated in the air part when the light travels through the hollow-core fibers, and only a small part of the light overlaps with the surrounding glass structure. These fibers can take advantage of the ultra-low Rayleigh scattering and nonlinearity coefficients of the air (one order of magnitude lower than any glass), allowing lasers to propagate at ultralow losses and nonlinearity. In addition, compared with all-solid-state core fibers, light travels 40% faster in hollow-core fibers than in traditional silica fibers, which can achieve nearly 99.7% of the speed of light in a vacuum, and the optical signal transmission speed per kilometer is accelerated by 1.54 μs, with lower time delay and a higher laser damage threshold [4,5,9,10,11]. In principle, compared with solid-state fibers, hollow-core fibers are less sensitive to environmental disturbances such as mechanical vibrations, magnetic fields, and ionizing radiation [9].

In general, two kinds of hollow-core optical fibers have been proposed according to the waveguide principle: The first is hollow-core photonic bandgap (HC-PBG) fiber, with multiple layers of periodic air pores arranged in the cladding. Light is confined inside the hollow core through the PBG effect [1]. Light in the bandgap cannot propagate in the periodic air pores of the fiber cladding but can only be bound to the core. In 2004, Manyan et al. [12] reported a kind of HC-PBG fiber with a loss of 1.7 dB/km at a wavelength of 1.62 μm, with a transmission bandwidth of 70 nm. In 2005, Roberts et al. [13] reduced the loss of the HC-PBG fiber in this band to 1.2 dB/km, and they pointed out that the loss was close to the limit due to the limitation of surface scattering loss (SSL). In addition to their limited transmission bandwidth, HC-PBG fibers exhibit considerable power overlap and high group delay dispersion (especially near bandgap edges) [14].

Another type of hollow-core fiber is usually referred to as hollow-core anti-resonant fiber (HC-ARF). In 2002, N.M. Litchinitser et al. [15] pioneered the use of the anti-resonant reflecting optical waveguide (ARROW) guiding mechanism, using glass walls to form a Fabry–Perot resonant cavity structure in the cladding. By controlling the incident wavelength and the thickness of the glass wall, the resonance and anti-resonance conditions can be controlled. When the anti-resonance condition is satisfied, the transmission of the resonant cavity is the least and the reflectivity is the most, and the light is restricted in the fiber core by reflection, thereby forming an optical waveguide.

For HC-ARF, because the contact surface between the core and the cladding quartz wall is small, and the core diameter size is large, the light field intensity is weak at the intersection, and the SSL can be reduced by an order of magnitude [9,14]. To control the modal content and attenuation coefficient, HC-ARF nested with several types of anti-resonant tubes has been proposed, studied, and manufactured, including circular anti-resonant tubes [4,10,16,17,18,19], “ice cream cone”-shaped anti-resonant tubes [19,20], elliptical anti-resonant tubes [21], nested anti-resonant tubes [5,9,11,22], and more complex shaped anti-resonant tubes [23]. In addition, HC-ARF has become one of the research hotspots in the field of optical fibers due to its advantages, such as ultrawide transmission bandwidth, low confinement loss, and relatively simple preparation. In 2022, Xin Zhang et al. [22] reported the preparation and characterization of a nested five-tube HC-ARF with an attenuation rate of 0.85 dB/km at 2 μm. The bandwidth was 200 nm with a confinement loss below 2 dB/km, and the HC-ARF showed excellent modal purity.

In this study, in order to obtain HC-ARF with better transmission performance in the 2 μm band, and to reduce the difficulty of drawing and obtaining a more easily implemented optical fiber design, several of the most traditional and mature HC-ARF structures were systematically studied to achieve the lowest transmission loss and the best single-mode performance, as well as a design that was insensitive to bending in the 2 μm band. Moreover, to obtain complete information about the modal content of the fiber, the confinement loss of the fundamental mode (FM) and higher-order modes (HOMs) under different geometric parameters, as well as the higher-order mode extinction ratio (HOMER), was studied. Our numerical study provides theoretical guidance for the correct selection of the number and size of the anti-resonant cladding tubes to achieve low transmission loss and high single-mode performance of the 2 μm laser.

## 2. Fiber Geometry

**Figure 1:**
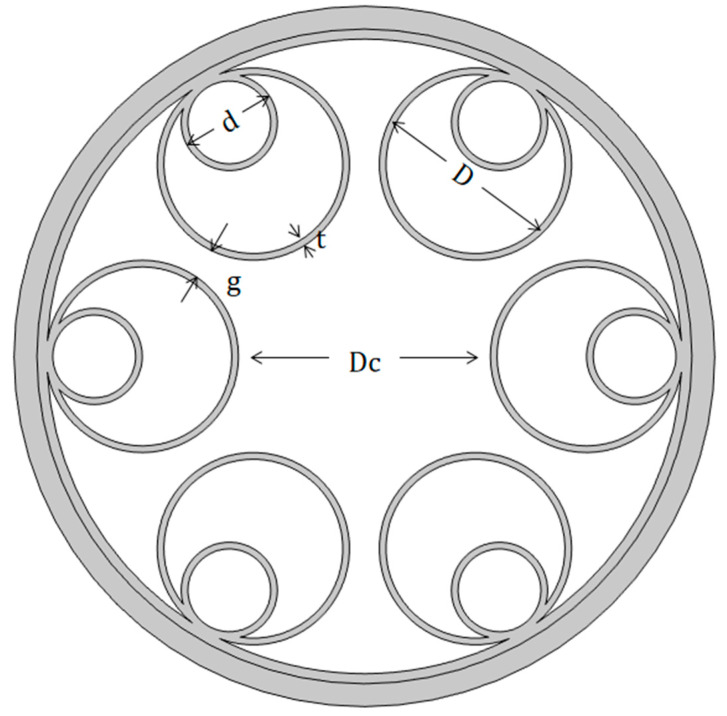
Schematic diagram of the structure of hollow-core nested anti-resonant fiber (HC-NANF or NANF).

Figure 1 shows the geometry of the six-tube nested HC-ARF that we analyzed, where $D_{c}$ is the core diameter, i.e., the diameter of the largest inner circle in the middle of the anti-resonant tube. The core radius of the HC-ARF has a crucial influence on the confinement loss. The loss is inversely proportional to the core diameter, but the way to obtain low loss by increasing $D_{c}$ is not without limitations, and the higher-order mode loss in the fiber will also decrease with the increase in $D_{c}$, which is harmful to the single-mode transmission of the optical fiber. *d* is the diameter of the inner anti-resonant tube, g is the gap distance between the anti-resonant tubes, and the contact points between the anti-resonant tubes are called nodes. Although the cladding of this fiber structure with nodes is more blocked and there is no gap, it has been shown that these nodes will produce Fano resonance when the light travels through the fiber, which increases the loss of the fiber and adversely affects the transmission of the fiber [24]. A nodeless design can provide better loss attributes. However, excessively large g will lead to light leakage. *D* is the diameter of the anti-resonant tube, and its numerical calculation formula is as follows:

(1) $$D=\frac{g-D_{c}\times \sin(\frac{\pi }{N})}{\sin(\frac{\pi }{N})-1},$$

where *N* is the number of anti-resonant cladding tubes, and *t* is the wall thickness of the anti-resonant tube. Its numerical calculation formula is as follows:

(2) $$t=\frac{\lambda_{m}\times \left(m-0.5\right)}{2\times \sqrt{n^{2}-1}},$$

where $\lambda_{m}$ is the wavelength, m is the resonance order (non-zero integer), and n is the refractive index of the cladding tube.

## 3. Numerical Results

For numerical calculations, we used the COMSOL mode solver with the finite element method. To accurately simulate the confinement loss of the fiber, the perfectly matched layers (PMLs) were adopted outside the fiber zone. To achieve accurate results, the mesh size of PML is critical in thin silica walls. Therefore, the fine mesh sizes of λ/4 and λ/6 were used in the sections of air and silica, respectively [9]. To ensure the convergence of the numerical results, the code was tested by reproducing the results in [22,25].

### 3.1. Parameter Optimization of Six-Tube Node-Free Nested HC-ARF

**Figure 2:**
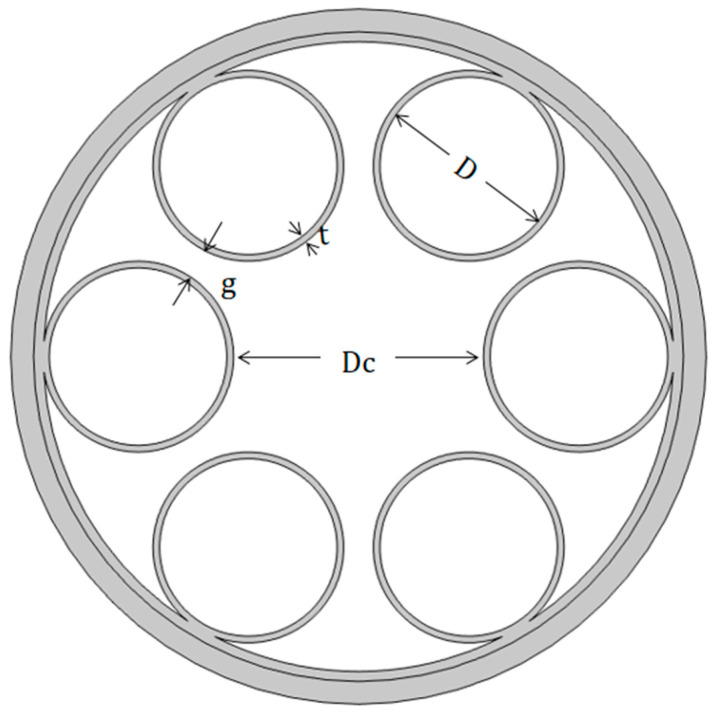
Structure diagram of HC-ARF with circular cladding tubes.

Figure 2 shows the schematic diagram of the classic six-tube HC-AR fiber, which has more confinement loss compared with classic six-tube nested HC-AR fiber, because it has one less layer of the anti-resonant tube. However, its simple construction and easy processing make it convenient for theoretical simulation. With only one layer of anti-resonant tube, the independent parameters that affect the fiber structure are only $D_{c},\;\;g,\;\mathrm{and}\;\;t$ when the number of tubes is six, and the diameter of the anti-resonant tube *D* can be determined by Equation (1) using the parameters $D_{c},\;\;g,\;\;\mathrm{and}\;N$.

**Figure 3:**
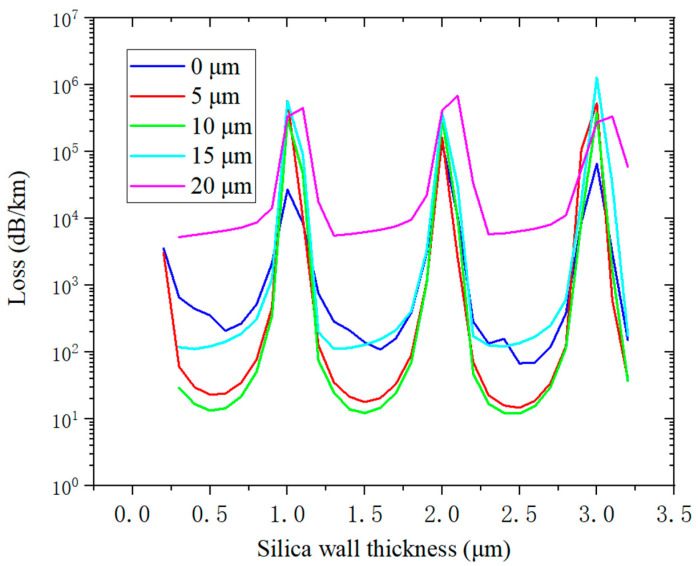
The curve of confinement loss with different silica wall thicknesses t and different gap distances g.

The number of cladding tubes (N) was six in this simulation; the gray area represents quartz, and its refractive index was determined by Sellmeier’s formula. The material represented by the white area is air, with a refractive index of 1.0. The wavelength was set to 2 μm. According to known research, the core diameter of optical fibers is generally set to be about 30 times the wavelength [7], so the core diameter $D_{c}$ was set to be 55 μm. Using Equation (2), it can be inferred that the first-order and the second-order anti-resonance thicknesses are 0.48 μm and 1.46 μm, respectively. In order to determine the best silica strut thickness and simulate the influence of optical fiber thickness on confinement loss, we calculated the confinement loss of the geometric model in Figure 2 with different silica wall thicknesses t and different gap distances g. The results are shown in Figure 3.

It can be seen from Figure 3 that there are three transmission regions in the wall thickness range of 0.3–3.2 μm. The centers of the first and the second transmission regions are at a wall thickness of 0.5 μm and 1.5 μm, respectively. This is consistent with the ARROW model. Different gap distances have little influence on the transmission region but have a great influence on the loss. Due to the inverse relationship between the resonant wavelength bandwidth and m, the larger the *m*, the narrower the corresponding bandwidth. When m = 1, the wall thickness is too thin. Thus, we chose t = 1.46 μm (when m = 2) as the thickness of the anti-resonant tube wall for the simulation.

**Figure 4:**
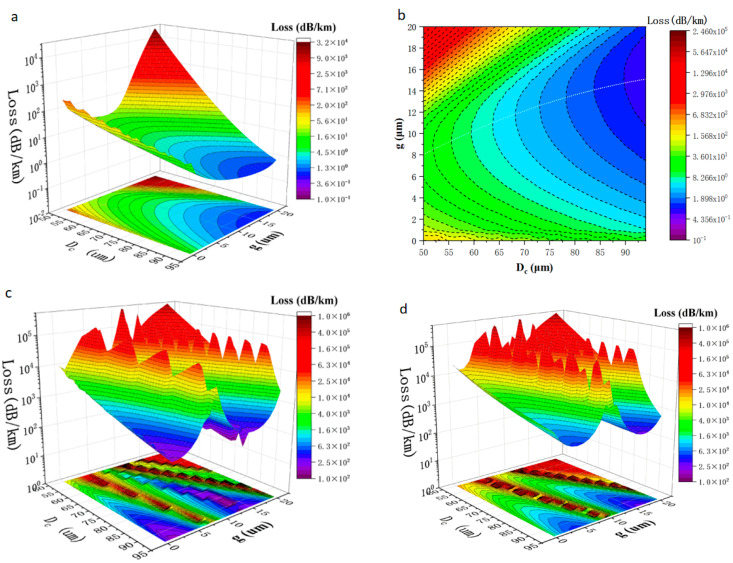

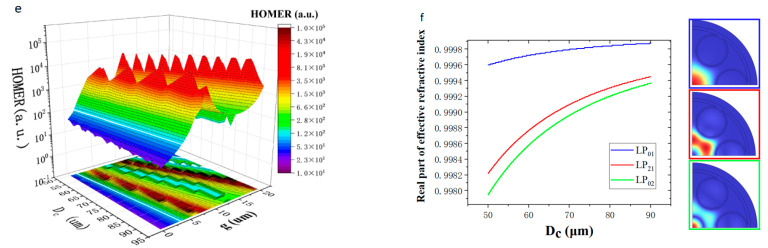
Different core diameters $D_{c}$ and gap distances g corresponding to different confinement losses and HOMERs: (**a**) Confinement loss of $LP_{01}$ fundamental mode (FM). (**b**) Confinement loss of $LP_{01}$ FM; the white dashed line represents the lowest confinement loss when the core diameter is different. (**c**) Confinement loss of $LP_{21}$ mode. (**d**) Confinement loss of $LP_{02}$ mode. (**e**) HOMER; the white line is the contour line when HOMER is 100. (**f**) The real part of the effective refractive index of $LP_{01},LP_{21\;}$, and $LP_{02}\;$ modes.

The core radius of an HC-ARF has a crucial influence on the loss, and it can even determine the loss magnitude of the optical fiber. Although the loss is inversely proportional to the diameter of the core, it is limited to attaining low loss by increasing $D_{c}$. With the increase in $D_{c}$, the loss of higher-order modes will also decrease, which is unfavourable for single-mode propagation, Therefore, $D_{c}$ is an important index for optimizing HC-ARF. Figure 4 shows the confinement losses and HOMER corresponding to different core diameters and different gap distances.

Figure 4a shows that the confinement loss decreases sharply with the increase in the core diameter, and the white dotted line in Figure 4b marks the lowest loss of different core diameters and shows different gap distances corresponding to different core diameters when the lowest loss occurs. Coincidentally, when the loss is the lowest, the ratio of the diameter of the anti-resonant tube to the diameter of the fiber core $D/D_{c}\;$happens to be about 0.66. As a comparison, when $D_{c}$ = 90 μm, the lowest confinement loss is 0.89 dB/km, while the loss reaches up to 20.85 dB/km at 50 μm. Although the simulation shows that a larger core diameter performs better in terms of confinement loss, the loss of the higher-order mode will also increase with the increase in the core diameter. Figure 4c shows the confinement loss of the $LP_{21}$ mode, which has the lowest loss in propagation compared with other higher-order modes. Figure 4e shows the calculated HOMER, which is defined as the ratio between the lowest confinement loss of the HOM and the confinement loss of FM [9,21]. Current studies suggest that the extinction ratio of hollow-core anti-resonant fiber to higher-order mode should be at least above 100 to have an ideal pseudo-single-mode propagation performance [26]; the white line is the contour line when HOMER is 100. From the figure, it can be seen that there is a wide range on both sides of the gap of the optimal anti-resonant tube, and no matter how large the diameter of the core is, good quasi-single-mode propagation can be achieved. Therefore, the decrease in HOMER is not the factor that limits the core diameter. Figure 4f shows the real part of the effective refractive index of $LP_{01},LP_{21\;}$, and $LP_{02}\;$ modes.

**Figure 5:**
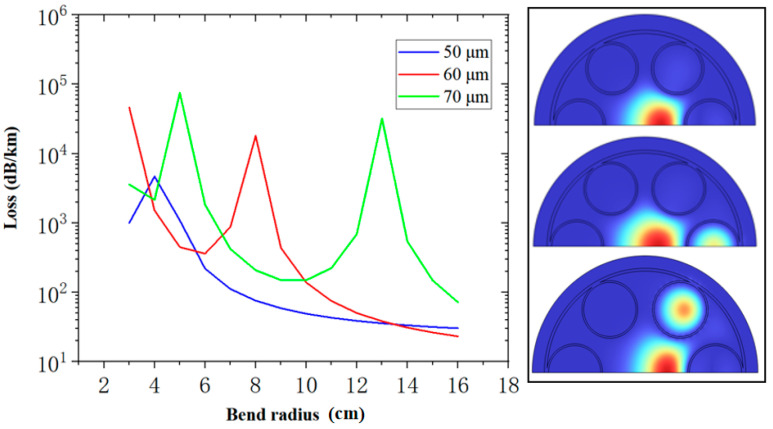
Relationship between bending loss and bending radius at 2 μm wavelength when the core diameters are 50 μm, 60 μm, and 70 μm. All fibers have the same $D/D_{c}=$ 0.66 and t=1.46 μm.

In addition to the increase in $D_{c}$ being affected by the decrease in HOMER, the bending loss is also a parameter to be considered when designing a fiber. Bending loss (BL) is caused by the partial mode energy escaping from the cladding when the fiber is bent. The calculation of BL needs to adopt the equivalent straight fiber method in simulation [27], and the refractive index of equivalent straight fiber will vary along the bending direction; this varied refractive index distribution can be described by Equation (3).

(3) $$n^{\prime }=n\cdot e^{\frac{x}{R_{c}}}\sim n\cdot \left(1+\frac{x}{R_{c}}\right),$$

where $R_{c}$ represents the bending radius, n is the refractive index distribution of the original optical fiber, and x represents the abscissa position of a point on the equivalent straight fiber (i.e., the distance from the center of the fiber core to the point). After the equivalence, the refractive index property of the material can be redefined in COMSOL. In this simulation, the material refractive index of the fiber model becomes a function of x, and then the effective refractive index is calculated by mode analysis. Because bending loss can be seen as a kind of light leakage, with the imaginary part of the effective refractive index, the corresponding bending loss can be calculated by the formula for calculating the confinement loss. The results are shown in Figure 5.

**Figure 6:**
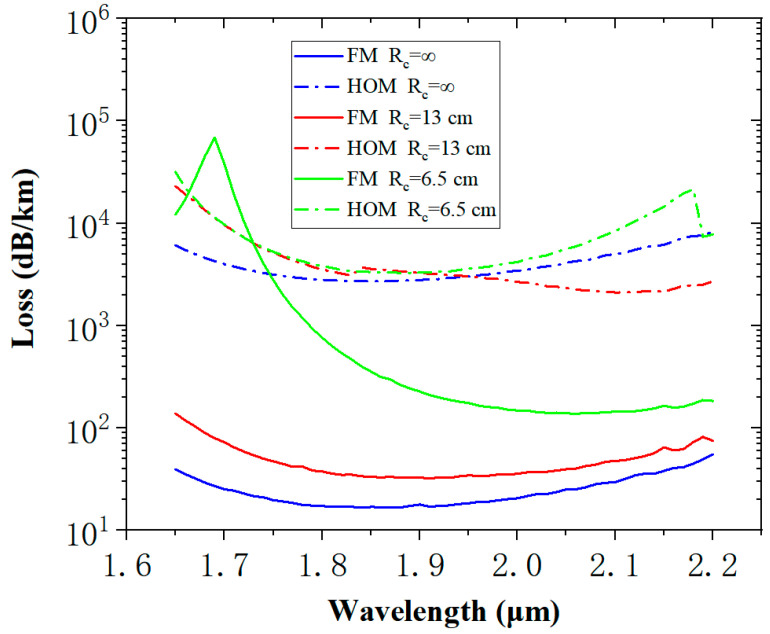
Diagram of the bending loss versus the wavelength in the HC-ARF. All fibers have the same core diameter$\;D_{c}$ = 50 µm, a uniform silica wall thickness t=1.46 μm, and$\;D/D_{c}=$ 0.66.

The loss peak observed in the figure is due to the mode coupling between the core and the cladding tube mode, as shown in the calculated modal field profile on the right-hand side. Although the simulation of straight fiber shows that the larger the core diameter, the lower the loss, the simulation of bending loss indicates that a larger core diameter leads to more bending loss. However, bending is inevitable in the normal operation of the optical fiber; thus, the core diameter should not be increased indefinitely. Figure 6 shows the confinement loss in fiber when $R_{c}$ = ∞, $R_{c}$ = 13 cm, and $R_{c}$ = 6.5 cm, while $D_{c}$ is kept to 50 µm.

### 3.2. Parameter Optimization of Six-Tube Node-Free Nested HC-ARF

**Figure 7:**
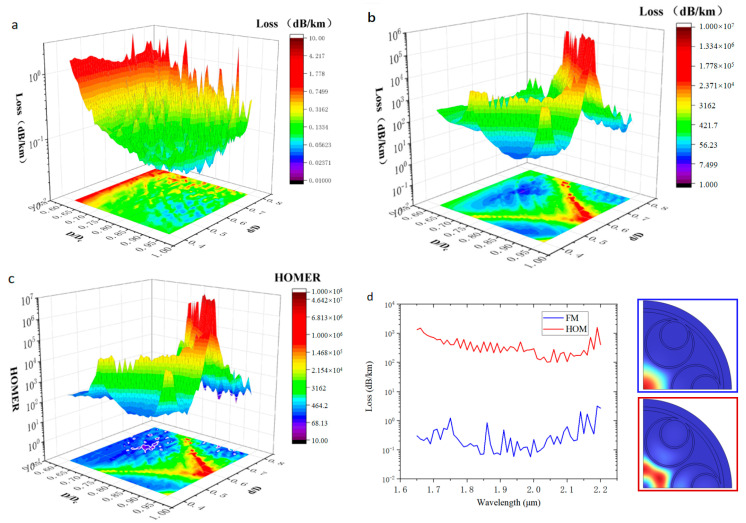

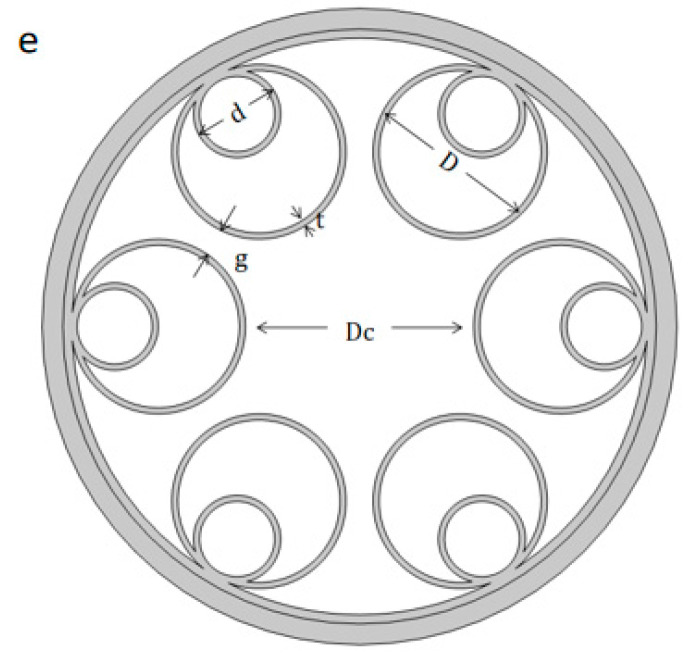
Diagram of confinement loss and extinction ratio of higher-order modes corresponding to different $D/D_{c}$ and different d/D: (**a**) Confinement loss diagram of $LP_{01}$ FM corresponding to different $D/D_{c}$ and different d/D. (**b**) Confinement loss diagram of HOM $LP_{21}$ corresponding to different $D/D_{c}$ and different d/D. (**c**) HOMER, where the white line is the contour line when HOMER is 100. (**d**) Calculated confinement loss spectrum of $D/D_{c}$ = 0.89, d/D = 0.6. (**e**) The geometry of a node-free nested HC-ARF with 6 anti-resonant tubes.

Hollow-core anti-resonant fibers guide light through anti-resonant reflection conditions, but this kind of cavity-like structure cannot show 100% reflectance—a small part of the light will still transmit through the glass wall, resulting in energy loss. Generally, adding a layer of the anti-resonant tube inside one layer of the tube to form a multilayer nested resonant cavity structure can significantly reduce the confinement loss. With the increase in the number of nested layers, the confinement loss will also be reduced, but the gain in terms of confinement loss is very small, which is accompanied by the complexity of the fiber structure [5]. Therefore, we set the number of nesting layers to one. According to the previous simulation, the confinement loss is correlated with the ratio of the anti-resonant tube’s diameter to the core diameter. Therefore, we show a contour plot of the FM and HOMs confinement loss of the six-tube node-free nested HC-ARF in Figure 7, where the wavelength is 2 μm$,D_{c}$ = 50 μm, and *t* = 1.46 μm, with the normalized anti-resonant tube diameter ($D/D_{c}$) and normalized inner anti-resonant tube diameter (d/D) as variables. From these contour plots, the regions with low confinement loss and single-mode transmission can be effectively determined.

The simulation results show that the six-tube node-free nested HC-ARF with a wavelength of 2 μm has the lowest confinement loss at $D/D_{c}$ = 0.89, d/D = 0.6, and the confinement loss is 0.042 dB/km. HOMER exceeds 100 in most parts; for $D/D_{c}$ = 0.89, d/D = 0.6, HOMER is more than 9000, and the HC-ARF has excellent single-mode transmission performance.

### 3.3. Parameter Optimization of Five-Tube Node-Free Nested HC-ARF

**Figure 8:**
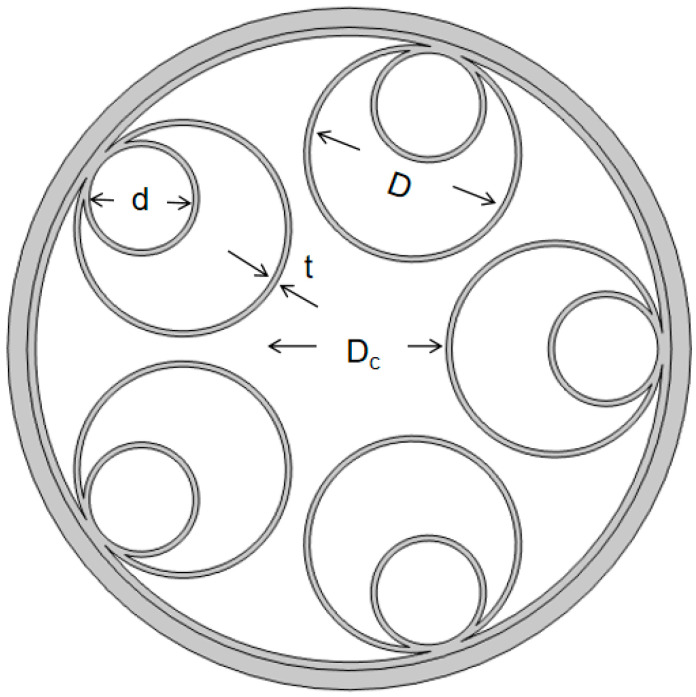
The geometric structure of a node-free nested HC-ARF with 5 anti-resonant tubes.

According to the theory of Habib et al. [25], the five-tube nested HC-ARF has lower confinement loss and a wider transmission window than the six-tube and seven-tube nested HC-ARF. Figure 8 shows the geometric structure diagram of the nested HC-ARF with five anti-resonant tubes.

**Figure 9:**
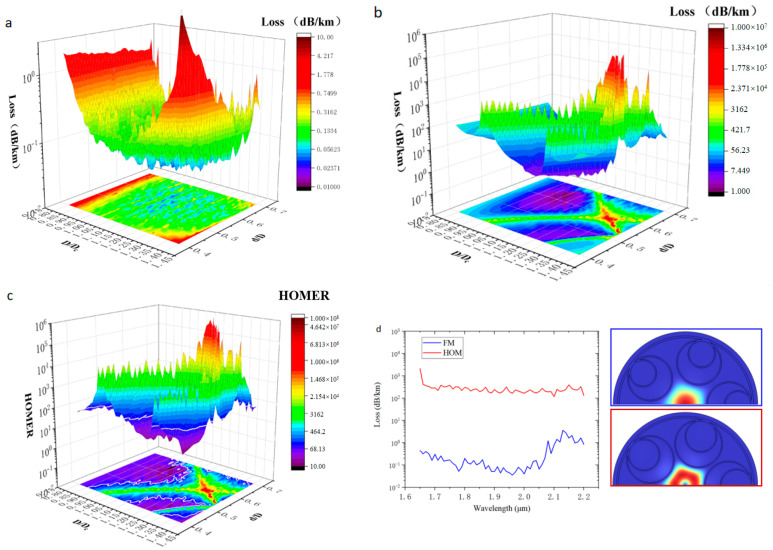
Diagram of confinement loss and extinction ratio of higher-order modes corresponding to different $D/D_{c}$ and different d/D: (**a**) Confinement loss diagram of $LP_{01}$ FM corresponding to different $D/D_{c}$ and different d/D. (**b**) Confinement loss diagram of higher-order mode $LP_{21}$ corresponding to different $D/D_{c}$ and different d/D. (**c**) HOMER, where the white line is the contour line when HOMER is 100. (**d**) Calculated confinement loss spectrum of $D/D_{c}$ = 1.12, d/D = 0.53.

We show contour plots of the FM and HOMs confinement losses of the five-tube node-free nested HC-ARF in Figure 9, where the wavelength is 2 μm, $D_{c}$ = 50 μm, t = 1.46 μm, and the normalized anti-resonant tube diameter ($D/D_{c}$) and the normalized nested bushing diameter (d/D) are taken as variables. From these contour plots, the regions with low confinement losses and effective single-mode transmission can be determined.

**Figure 10:**
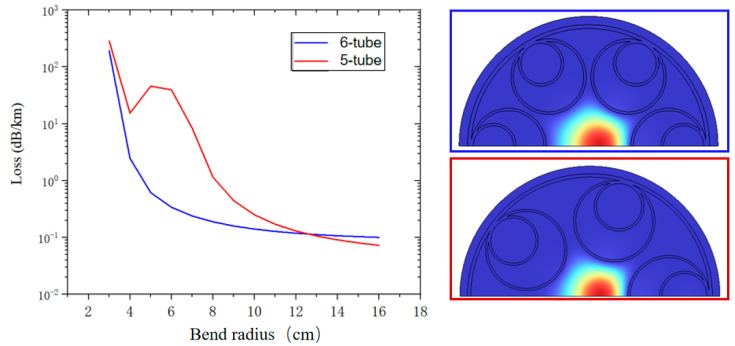
Confinement loss at the wavelength of 2 μm with different bending radii.

The five-tube nested HC-ARF has lower confinement loss than the six-tube nested HC-ARF. However, with the decrease in the number of anti-resonant tubes, the anti-resonant tube diameter and the area of the air in the tube will also increase. In few-tube designs, the ultra-large area of air in the anti-resonant tube will result in the same or even a higher effective refractive index compared with that of the core, resulting in higher FM confinement loss [9] and much higher bending loss—that is, mode coupling between the core and the cladding tube mode is more likely to occur. A smaller pseudo-single-mode output region is shown in this simulation, as presented in Figure 9c, where the white line is a contour line with the HOMER of 100, such as the HOMER diagram in Figure 7c obtained by the nested HC-ARF with six tubes. The region of HOMER greater than 100 is much smaller. At the lowest point of FM confinement loss when $D/D_{c}$ = 1.07, d/D = 0.61, the confinement loss of the wavelength at 2 μm is 0.035 dB/km, but HOMER is only 86.5, which does not meet the condition of the pseudo-single-mode transmission. However, when $D/D_{c}$ = 1.12, d/D = 0.53, the confinement loss of the wavelength at 2 μm is 0.040 dB/km, and HOMER is >2700. Therefore, a five-tube structure can obtain better confinement loss compared with that of a six-tube structure, but the optimal parameters of the fiber used need to be more accurate and sensitive—that is, the design with minimum confinement loss cannot be easy to adopt. Figure 10 shows the confinement loss of five-tube ($D/D_{c}$ = 1.12, d/D = 0.53) and six-tube ($D/D_{c}$ = 0.89, d/D = 0.6) structures at different bending radii. When the radius is less than 12 cm, the confinement loss of the five-tube structure is greater than that of the six-tube structure, indicating that the larger air area in the anti-resonant tube has a negative effect on the confinement loss during bending. When the radius is greater than 12 cm, the confinement loss of a five-tube structure is less than that of a six-tube structure, with the five-tube structure having lower confinement loss than that of the six-tube structure in the straight fiber.

## 4. Conclusions

In this paper, to obtain HC-ARF with better transmission performance in the 2 μm band, and to reduce the difficulty of drawing and obtaining a more easily implemented optical fiber design, we determined the design of the mature and traditional five-tube and six-tube node-free nested HC-ARF, with the features of confinement loss < 0.05 dB/km, low bending confinement loss, and single-mode propagation. To obtain the complete parameter information of the optical fiber modal content, we analyzed and optimized the confinement loss and single-mode characteristics by carefully adjusting the normalized anti-resonant tube diameter ($D/D_{c}$) and the normalized inner tube diameter (d/D). We proved that the details of the cladding structure and the number of anti-resonant cladding tubes significantly affected the confinement loss and single-mode operation. We found that the six-tube node-free nested HC-ARF had the lowest confinement loss at $D/D_{c}$ = 0.89 and d/D = 0.6, with a confinement loss of 0.042 dB/km, HOMER > 9000, and excellent single-mode transmission performance. Moreover, we found that the five-tube node-free nested HC-ARF had a wider transmission bandwidth and lower confinement loss compared with the six-tube system, while the region of HOMER greater than 100 was much smaller, and the bending loss was also larger. Therefore, although a five-tube HC-ARF can obtain better confinement loss compared with that of a six-tube system, the optimal parameters that the HC-ARF used need to be more accurate and sensitive—that is, the design with minimum confinement loss cannot be easy to adopt. Our numerical study provides theoretical guidance for correctly selecting the number and size of anti-resonant cladding tubes to achieve low confinement loss and high single-mode propagation performance in the 2 μm region.

## Data Availability

Data are available upon request from the corresponding author.
